# Synaptic Endocannabinoid Signaling in the Anterior Cingulate Cortex: Implications for Alzheimer's Disease Pathology and Social Behavior

**DOI:** 10.1002/alz70855_097499

**Published:** 2025-12-23

**Authors:** Jiali Li

**Affiliations:** ^1^ The Neuroscience Institute at JFK Medical Center, Edison, NJ, USA

## Abstract

**Background:**

Alzheimer's disease (AD) is a major contributor to neuropsychiatric disorders, exerting profound impacts on individuals and society. Social behavioral impairments associated with AD present significant challenges for both diagnosis and treatment, highlighting the urgent need to unravel their underlying mechanisms. Dysfunction of the anterior cingulate cortex (ACC) has been identified as a key factor driving the emergence of these behavioral deficits. Among its regulatory mechanisms, endocannabinoids play a critical role in modulating short‐term synaptic plasticity in the ACC, thereby maintaining synaptic homeostasis. Endocannabinoid signaling is highly sensitive to environmental stimuli, demonstrating dynamic responses to external stressors. Despite these insights, the precise role of synaptic endocannabinoid signaling in the ACC, particularly its contribution to synaptic homeostasis and social behavioral regulation in the context of AD pathology, remains poorly understood.

**Method:**

Using a multifaceted approach—including optogenetic, electrophysiological, pharmacological, and behavioral techniques—we characterized alterations in presynaptic CB1 receptors and endocannabinoid synthesis at excitatory and inhibitory synapses during AD progression.

**Result:**

Our findings reveal a regulatory role of cannabinoid signaling at both pre‐ and post‐synaptic terminals within the ACC, providing insights into its modulation of synaptic transmission in AD. We further examined the impact of disrupted endocannabinoid signaling on synaptic homeostasis, employing calcium signal recording and pharmacological interventions. Alterations in excitatory and inhibitory synaptic function were particularly evident in socially isolated mice, a condition that exacerbates AD‐related behavioral deficits. These findings highlight the interplay between endocannabinoid dysregulation, synaptic dysfunction, and behavioral abnormalities in AD. Moreover, we explored therapeutic strategies targeting synaptic endocannabinoid signaling to mitigate AD‐induced social behavioral deficits. Using cannabinoid receptor knockout models and pharmacological approaches, we dissected the distinct roles of cannabinoid signaling components in mediating behavioral outcomes. This work underscores the potential of modulating endocannabinoid signaling to alleviate neuropsychiatric symptoms associated with AD.

**Conclusion:**

This comprehensive investigation sheds light on the intricate relationship between AD pathology, synaptic endocannabinoid signaling, and social behavior. By unraveling the molecular, cellular, and behavioral correlates of AD‐induced alterations in cannabinoid signaling, our study provides valuable insights into the pathophysiology of AD‐related neuropsychiatric disorders. It lays the foundation for innovative therapeutic approaches.

